# Which neuroimaging and fluid biomarkers method is better in theranostic of Alzheimer’s disease? An umbrella review

**DOI:** 10.1016/j.ibneur.2024.02.007

**Published:** 2024-02-29

**Authors:** Hossein Mohammadi, Armin Ariaei, Zahra Ghobadi, Enam Alhagh Charkhat Gorgich, Auob Rustamzadeh

**Affiliations:** a Department of Bioimaging, School of Advanced Technologies in Medicine, Isfahan University of Medical Sciences (MUI), Isfahan, Islamic Republic of Iran; b Student Research Committee, School of Medicine, Iran University of Medical Sciences, Tehran, Islamic Republic of Iran; c Advanced Medical Imaging Ward, Pars Darman Medical Imaging Center, Karaj, Islamic Republic of Iran; d Department of Anatomy, School of Medicine, Iranshahr University of Medical Sciences, Iranshahr, Islamic Republic of Iran; e Cellular and Molecular Research Center, Research Institute for Non-communicable Diseases, Qazvin University of Medical Sciences, Qazvin, Iran

**Keywords:** Alzheimer’s disease, Cerebrospinal fluid, Neuroimaging, Biomarkers, Magnetic resonance imaging, Plasma, Diagnosis

## Abstract

Biomarkers are measured to evaluate physiological and pathological processes as well as responses to a therapeutic intervention. Biomarkers can be classified as diagnostic, prognostic, predictor, clinical, and therapeutic. In Alzheimer’s disease (AD), multiple biomarkers have been reported so far. Nevertheless, finding a specific biomarker in AD remains a major challenge. Three databases, including PubMed, Web of Science, and Scopus were selected with the keywords of Alzheimer’s disease, neuroimaging, biomarker, and blood. The results were finalized with 49 potential CSF/blood and 35 neuroimaging biomarkers. To distinguish normal from AD patients, amyloid-beta_42_ (Aβ_42_), plasma glial fibrillary acidic protein (GFAP), and neurofilament light (NFL) as potential biomarkers in cerebrospinal fluid (CSF) as well as the serum could be detected. Nevertheless, most of the biomarkers fairly change in the CSF during AD, listed as kallikrein 6, virus-like particles (VLP-1), galectin-3 (Gal-3), and synaptotagmin-1 (Syt-1). From the neuroimaging aspect, atrophy is an accepted biomarker for the neuropathologic progression of AD. In addition, Magnetic resonance spectroscopy (MRS), diffusion weighted imaging (DWI), diffusion tensor imaging (DTI), tractography (DTT), positron emission tomography (PET), and functional magnetic resonance imaging (fMRI), can be used to detect AD. Using neuroimaging and CSF/blood biomarkers, in combination with artificial intelligence, it is possible to obtain information on prognosis and follow-up on the different stages of AD. Hence physicians could select the suitable therapy to attenuate disease symptoms and follow up on the efficiency of the prescribed drug.

## 1 Introduction

Alzheimer’s disease (AD), a well-known neurodegenerative disease, is widely perceived as amyloid-beta (Aβ) accumulation and neurofibrillary tangles (NFT) located in the brain parenchyma, resulting in synaptic malfunction and memory loss (Lashley et al., 2018, Shah-Abadi et al., 2023). Up to this date, AD diagnosis encounters several challenges and dubiety, due to contradictory anticipation of molecular science versus clinical aspects. In this regard, biomarkers could play a crucial role in facilitating AD diagnosis. The cerebrospinal fluid (CSF) biomarkers depict high sensitivity to AD prognosis, since any changes in the neuron properties including degeneration, could be immediately reflected in the CSF (Moghekar et al., 2013).

In the cerebrospinal fluid (CSF) of AD patients, measurements of Aβ1–42 (Aβ42), total tau (tTau), and phosphorylated tau at Thr181 (pTau181) have proven useful in the early diagnosis of AD, which leads to their inclusion in diagnostic guidelines (Dubois et al., 2014, McKhann et al., 2011). Current clinical surveys use measurements of Aβ and tau proteins in CSF and/or blood to guide participant recruitment and outcome measures (Niklas Mattsson et al., 2015). This practice allows for the inclusion of patients with AD pathology, even in the preclinical stage, and enables the monitoring of therapeutic efficacy on Aβ- and tau pathology. However, the usefulness of Aβ biomarkers for monitoring disease trends and drug response is limited due to early saturation of Aβ accumulation in the brain, which is indicated by plateaus in CSF Aβ42 levels and amyloid PET uptake after clinical symptom onset (Bateman et al., 2012, Le Bastard et al., 2013; Toledo et al., 2013). On the other hand, though tau protein levels are initially more indicative of the clinical status than Aβ, their correlations are also diminished as neurodegeneration advances, indicating a stabilization or decrease in protein levels (Sutphen et al., 2018, Toledo et al., 2013). The shortage of Aβ and tau biomarkers is a significant challenge during repeated failures of Aβ-targeting drug trials.

Besides the CSF, neuroimaging techniques could provide a true picture of AD diagnosis. Among a wide range of neuroimaging applications, magnetic resonance imaging (MRI) is reported to offer more data concerning AD diagnosis, as well as the determination of the disease stage. One of the hallmarks of AD detection in MRI is the existence of hippocampal atrophy (Albert et al., 2011, McKhann et al., 2011). Instead of evaluating a specific area of the brain, it is possible to have a wider concept of disease progression, especially in the later stages of AD, with the examination of the brain’s white and gray matters by performing T_1_-weighted sequences (Jack Jr et al., 2018, Kehoe et al., 2014). Nevertheless, researchers tend to detect AD more specifically, by focusing on diffusion tensor imaging (DTI), in which analyzing water diffusion along with their directional information is applied to assess brain white matter (WM) integrity. Moreover, detailed information on axon bundle orientation and changes in fiber tracts can be monitored with high efficiency (Mohtasib et al., 2022). One of the limitations of the mentioned methods is the inadequate data across the real-time function of neurons and their condition however, with the aid of positron emission tomography (PET) and functional MRI (fMRI), this phenomenon has been partly compensated. For instance, the application of radioligands could reveal targeted particles through PET scans and give the physician the ability to detect neuron metabolism, pathological protein accumulation, and the function of neuroreceptors (Tiepolt et al., 2019; Y. T. Wang & Edison, 2019). The success of the suggested methods in preclinical and limited sample size human research is mainly justified in the late stage of AD. It means that distinguishing mild cognitive impairment (MCI) and differentiating it from other diseases, still faces multiple limitations. In this review article, there was a focus on AD biomarkers in detection, follow-up, and discovering the pathological pathways involved in the disease, in order to develop potential drugs for the cessation of the AD cascade (Liu et al., 2016; Liu et al., 2018; Pan et al., 2020).

## 2 Materials and methods

### 2.1 Eligibility criteria

This study comprised the retrospective, prospective, clinical trial, cross-sectional, meta-analysis and review studies published the findings of fluid or neuroimaging biomarkers for AD in peer-reviewed journals. There was a restriction in the sample size of the original studies in which studies classified as case reports or case controls with less than 10 participants were excluded. In addition, studies labeled as conference proceedings, letters to the editor, lectures, and news were not included in this study. There was an attempt to list potential fluid and neuroimaging biomarkers in prediction, prognosis, or diagnostic approaches while excluding biomarkers in which the authors claimed further studies were conducted to confirm their results.

### 2.2 Search strategy and databases

PubMed, ScienceDirect, and Web of Science were selected as the search databases. The search strategy was based on the relevant keywords in the title and abstract of the articles including Alzheimer's disease, cerebrospinal fluid, biomarkers, serum, plasma, and neuroimaging. Moreover, to better select the relevant article, in the method section, imaging methods such as DTI and fMRI along with biomarkers detection tools in the plasma such as ELISA and mass spectrometry are considered. Finally, the following search strings were used to explore relevant articles in the selected databases.

•In the PubMed database: ((Alzheimers disease [Title/Abstract] AND biomarker[Title/Abstract]) AND (CSF[Title/Abstract] OR serum[Title/Abstract] OR plasma[Title/Abstract] OR neuroimaging[Title/Abstract])))

•In Web of Science and ScienceDirect databases: ((Alzheimer's disease AND biomarker) AND (CSF OR serum OR plasma OR neuroimaging))

### 2.3 Study selection

At first, there was a screening of the language of articles in which non-English articles were omitted from further evaluation. Subsequently, the inclusion criteria, mentioned in the eligibility criteria section, were applied to the identified articles. In the next step, the title and abstract of the articles were evaluated by three reviewers (AA, HM, and MB). Subsequently, the original articles’ methodology was reviewed from multiple aspects, including sample size, statistical analysis, and the material and tools used to determine the biomarkers level. In the case of unavailability of the article’s full text, the article was excluded from the study.

### 2.4 Data extraction

The summary of the data mentioned in the articles was extracted into a table by two researchers (AR and AA). The fluid biomarker was reported in a separate table from neuroimaging biomarkers.

### 2.5 Quality assessment

In order to exclude original articles with unclear or uncertain results and review articles that mentioned unreliable findings, the critical appraisal (CA) was utilized as a questionnaire assessment tool to define the risk of bias for the selected studies with the cooperation of two of the reviewers (AR and AA). Accordingly, the Standards for Reporting of Diagnostic Accuracy (STARD) checklist was utilized for the original studies mentioning biomarkers for AD.

## 3 Results

**Fig. 1:**
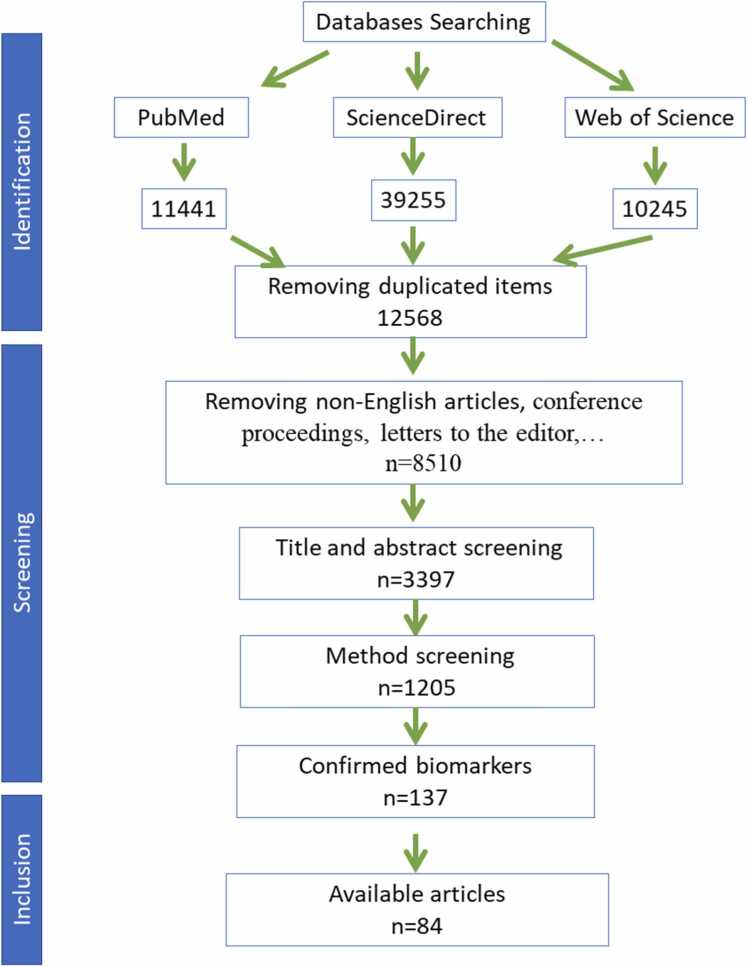
Flow diagram of the included studies. Subsequent to the searching in the databases, initial screening was applied to exclude irrelevant studies with the aim of the current study which explored AD biomarkers. In addition to initial screening, the methodology of the studies was evaluated to exclude studies with insufficient data including sample size, analytic tools, etc. Finally, a total number of 84 articles reporting the diagnostic value of AD fluid or neuroimaging biomarkers were included.

After removing the duplicated items, the outcome was concluded in 49 potential CSF/blood and 35 neuroimaging biomarkers (Fig. 1).

### 3.1 Blood and CSF biomarkers

#### 3.1.1 CSF biomarkers

Aβ is the most known CSF biomarker in AD, which is derived from a transmembrane protein called amyloid precursor protein (APP), with two amino terminals located in extracellular space and carboxyl located in intracellular space (Haass et al., 2012). APP can interact with either α-secretase or β-secretase, in which the first enzyme produces sAPPα (H. Wang et al., 2021), while the second one produces sAPPβ (Chasseigneaux & Allinquant, 2012). Aβ has multiple forms based on its peptide length, named as Aβ38, Aβ40, and Aβ42. The level of each of these forms could be used to distinguish AD from other neurodegenerative diseases. In a study by Olsson et al., the author rejects the application of sAPPα, sAPPβ, and Aβ38, in AD prognosis (Olsson et al., 2016). In a recent study, Cullen et al. declared a correlation between the amounts of CSF Aβ38 levels and AD stages, since patients with higher values have less chance of getting the disease (Cullen et al., 2022). In contradiction, two articles claimed elevated amounts of Aβ38 and Aβ40 in the CSF of AD patients (N. Mattsson et al., 2016; Tijms et al., 2018). The results of these studies reflected a significant change in the level of different forms of Aβ. Nevertheless, defining the changes to diagnose AD is still controversial. It is worth mentioning that the level of these forms of Aβ changes in multiple neurodegeneration diseases. As illustrated in three pieces of research, the decrease in Aβ40 may reflect cerebral amyloid angiopathy (CAA), while the reduction in the Aβ38 and Aβ40, reflects frontotemporal dementia (FTD) (Chen et al., 2018, Gabelle et al., 2011, Heywood et al., 2018). To better differentiate neurodegenerative diseases Janelidze et al. utilized the Aβ42/Aβ40 and Aβ42/Aβ38 instead of each form solely. With the aid of this technique, different types of dementias like Parkinson's disease (PD) and Lewy bodies, could be distinguished from each other (Janelidze et al., 2016).

Total-tau (T-tau), as one of the markers of neurodegenerative disease, has the highest sensitivity to be detected in CSF and the value is amplified in pathological conditions (Blennow & Hampel, 2003). This biomarker lacks the ability to specifically distinguish AD since the amount of T-tau is elevated in a wide range of neurodegenerative diseases (Ariaei and Ramezani, 2023, Hesse et al., 2001, Zetterberg et al., 2006). Nevertheless, it is still useful in the detection of high-intensity neurodegenerative diseases such as Creutzfeldt–Jakob, with a 10–20 fold increase in the CSF (Riemenschneider et al., 2003, Skillbäck et al., 2014).

In a systematic review containing a meta-analyze, three novel AD biomarkers defined as β-2-glycoprotein, ApoA-1, and α-2-macroglobulin (α2 M), were reported to be detected in the CSF (Rehiman et al., 2020). Furthermore, using the Elisa kit, YKL-40 (chitinase-3 like-1, human cartilage glycoprotein-39, and chondrex) and neurogranin (Ng) levels could be determined in the CSF (Hampel et al., 2018).

#### 3.1.2 Plasma/serum

Aβ could be detected with high sensitivity in the plasma. Accordingly, Chen et al. suggested that the assessment of the plasma level of Aβ42, leads to the diagnosis of AD with the sensitivity and specificity of 88 % and 81 %, respectively (Olsson et al., 2016). The results of another research were in line with Chen et al., which illustrated the soluble forms of Aβ as a promising biomarker for AD detection (Meng et al., 2019). There is a wide range of biomarkers involved in AD, but only a few of them correlate with the pathological pathway of the disease. Among them, Neuron-specific enolase (NSE), heart fatty acid binding protein (HFABP), Virus-like particles-1 (VLP-1), and YKL-40 are reported to be elevated in plasma (Olsson et al., 2016). α2 M could be detected in the serum or plasma of AD patients either by implementing mass spectrometry or gel-based platforms (Rehiman et al., 2020). pancreatic polypeptide (PP) was an indirect indicator of the AD pathology, in which the plasma level was determined through Myriad RBM-Luminex xMAP-190 (Hu et al., 2012), Myriad RBM-Luminex xMAP-146 analytes (Nazeri et al., 2014), Myriad RBM-Luminex xMAP-151 analytes (Doecke et al., 2012), and Slow Off-rate Modified Aptamer (SOMAmer)–“SOMAscan”-1001 analyte (Sattlecker et al., 2014).

#### 3.1.3 CSF and plasma

Another form of tau protein that can be detected in both CSF (short fragments) and plasma, is Phosphorylated-tau (P-tau). The results derived from Meredith et al. research depicted an uncertainty in the diagnosis of AD based on plasma P-tau (Meredith Jr et al., 2013). On the contrary, Tan et al. demonstrated a high sensitivity and specificity in the detection of AD, with a rate of 85 % and 97 %, respectively (Tan et al., 2014).

Neurofilament light (NFL) seems to be a potential biomarker in distinguishing AD. NFL is a part of a three-membered group of neurofilaments characterized based on their weight, into light, medium, and heavy which together determine the size of the axon and its conductivity (Olsson et al., 2016). This biomarker can be measured in either the CSF or blood, by applying the ELISA technique with higher sensitivity in the detection of AD, compared to Aβ and tau (Gisslén et al., 2016). In a systematic review with an evaluation of twenty-three articles, it was declared that NFL has a significantly higher concentration in AD patients, in comparison to the normal group (Qu et al., 2021).

**Fig. 2:**
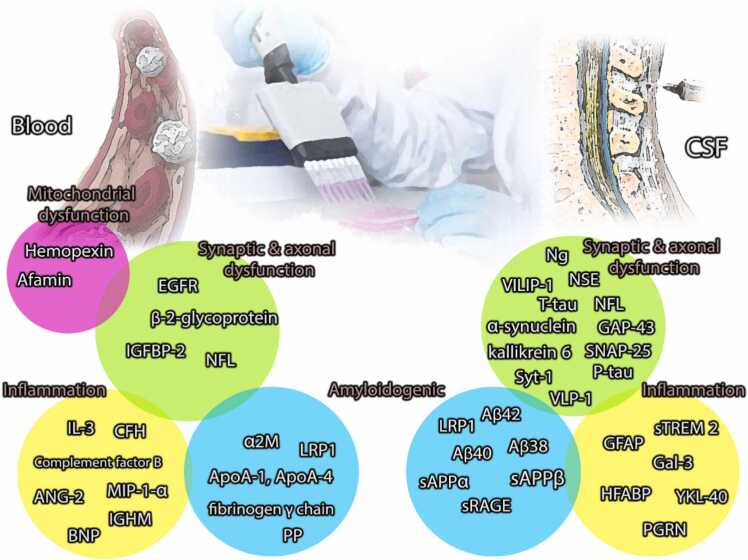
The fluid biomarkers of different pathways involved in AD are divided into blood and CSF based on their detection sensitivity. Furthermore, biomarkers were classified into four main groups based on the pathophysiology of AD, including synaptic and axonal dysfunction, inflammation, amyloidogenic, and mitochondrial dysfunction.

Other non-specific biomarkers reported to have significant level alternation in CSF and blood, are listed as: soluble triggering receptors expressed on myeloid cells 2 (sTREM 2), α-synuclein, and Progranulin (PGRN) (Qu et al., 2021) (Table 1) (Fig. 2).

**Table 1:** CSF and blood biomarkers in AD.

Row	Biomarker	Type of Research	Method	Highlighted	Sample	Reference
1	Aβ38	In vivo-human	2D-UPLC-tandem mass spectrometry	Can be used as a baseline to measure Aβ_42_	CSF	(Tijms et al., 2018)
2	Aβ40	In vivo-human	2D-UPLC-tandem mass spectrometry	Can be used as a baseline to measure Aβ_42_	CSF	(Tijms et al., 2018)
3	Aβ42	Meta-analysis	142 research from 1995 to 2014	Significantly higher level in AD compared to normal	CSF	(Olsson et al., 2016)
4	Neuron-specific enolase (NSE)	Meta-analysis	Total record of 4521 from PubMed and Web of Science	Higher level in AD compared to normal but less specifically toward other dementia diseases	CSF	(Hao et al., 2022)
5	heart fatty acid binding protein (HFABP)	Meta-analysis	5 research from 2011 to 2013	Significantly higher level in AD compared to normal	CSF	(Olsson et al., 2016)
6	Virus-like particles-1 (VLP-1)	Meta-analysis	Total record of 4521 from PubMed and Web of Science	Higher level in AD compared to normal but less specifically toward other dementia diseases	CSF	(Hao et al., 2022)
7	YKL-40 (chitinase-3 like-1, human cartilage glycoprotein-39, and chondrex)	In vivo- human	sandwich ELISA method (NF-light ELISA kit; Umandiagnostics AB, Umea, Sweden)	The level increased in the AD patients measured in nanograms	CSF	(Hampel et al., 2018)
8	β-2-glycoprotein	Meta-analysis	3 research from 2012 to 2014	2 studies reported a reduction in the plasma- 1 research reported an increasing level in the plasma	plasma	(Rehiman et al., 2020)
9	Apolipoprotein A-1 (ApoA-1)	Meta-analysis	3 research from 2012 to 2014	3 studies reported a reduction in the level of the plasma	plasma	(Rehiman et al., 2020)
10	Apolipoprotein A-4 (ApoA-4)	Meta-analysis	3 research from 2012 to 2017	2 studies reported increased levels in the plasma- 1 research reported decreasing levels in the plasma	plasma	(Rehiman et al., 2020)
11	pancreatic polypeptide (PP)	Meta-analysis	4 research from 2012 to 2014	all studies reported increased levels in the plasma	plasma	(Rehiman et al., 2020)
12	Afamin	Meta-analysis	3 research from 2012 to 2014	all studies reported increased levels in the plasma	plasma	(Rehiman et al., 2020)
13	Hemopexin	Meta-analysis	3 research from 2012 to 2014	all studies reported increased levels in the plasma	plasma	(Rehiman et al., 2020)
14	interleukin 3 (IL-3)	Meta-analysis	2 research from 2007 to 2012	all studies reported increased levels in the plasma	plasma	(Rehiman et al., 2020)
15	insulin-like growth factor binding protein-2 (IGFBP-2)	Meta-analysis	2 research from 2012 to 2014	all studies reported increased levels in the plasma	plasma	(Rehiman et al., 2020)
16	macrophage inflammatory protein- 1-α (MIP-1-α)	Meta-analysis	2 research from 2012 to 2013	all studies reported increased levels in the plasma	plasma	(Rehiman et al., 2020)
17	fibrinogen γ chain	Meta-analysis	3 research from 2012 to 2017	all studies reported increased levels in the plasma	plasma	(Rehiman et al., 2020)
18	α-2-macroglobulin (α2 M)	Meta-analysis	6 research from 2006 to 2014	3 studies reported increased levels in the plasma- 2 research reported increasing levels in the serum- 1 research reported a reduction in level in the serum	Plasma/ serum	(Rehiman et al., 2020)
19	angiopoietin-2 (ANG-2)	Meta-analysis	2 research from 2007 to 2013	all studies reported increased levels in the plasma	plasma	(Rehiman et al., 2020)
20	epidermal growth factor receptor (EGFR)	Meta-analysis	2 research from 2008 to 2012	all studies reported a reduction in the level of the plasma	plasma	(Rehiman et al., 2020)
21	Hemopexin	Meta-analysis	2 research from 2012	all studies reported increased levels in the plasma	plasma	(Rehiman et al., 2020)
22	complement factor H (CFH) precursor	Meta-analysis	2 research from 2006 to 2012	all studies reported increased levels in the plasma	plasma	(Rehiman et al., 2020)
23	Complement factor B	Meta-analysis	2 research from 2012 to 2015	all studies reported a reduction in the level of the plasma	plasma	(Rehiman et al., 2020)
24	brain natriuretic peptide (BNP)	Meta-analysis	2 research from 2012 to 2014	all studies reported increased levels in the plasma	plasma	(Rehiman et al., 2020)
25	soluble triggering receptor expressed on myeloid cells 2 (sTREM 2)	In vivo- human	Immunohistochemistry	Increase in the CSF of AD	CSF	(Boza-Serrano et al., 2022)
26	α-synuclein	In vivo-human	mass spectrometry	Increase in the CSF of AD	CSF	(Giannisis et al., 2022)
27	Progranulin (PGRN)	In vivo-human	Adipogen, Inc. (Catalog number AG-45A-0018YEK-KI01)	No differences in Lewy body dementia and AD compared to normal subject	CSF	(Morenas-Rodríguez et al., 2019)
28	sAPPα	In vivo- human	MSD sAPP-α/sAPP-β duplex assay (Meso Scale Diagnostics)	No significant difference between AD and normal	CSF	(Kettunen et al., 2022)
29	sAPPβ	In vivo- human	MSD sAPP-α/sAPP-β duplex assay (Meso Scale Diagnostics)	No significant difference between AD and normal but significant reduction in subcortical small-vessel disease	CSF	(Kettunen et al., 2022)
30	T-tau	In vivo human	multiplex xMAP Luminex platform (Luminex Corp, Austin, TX) Innogenetics (INNO-BIA AlzBio3, Ghent, Belgium;	Obstructive sleep apnea increases amyloid deposition, CSF T-tau, and P-tau levels over time, both in NL and MCI individuals.	CSF	(Bubu et al., 2019)
31	P-tau	In vivo-human	western-blotting	increase in CSF of AD patients	CSF	(Meredith Jr et al., 2013)
32	NFL	In vivo-human	sandwich ELISA method (NF-light ELISA kit; Umandiagnostics AB, Umea, Sweden)	The level significantly increased in the AD patients compared to MCI and normal measured in nanogram	CSF/ blood	(Hampel et al., 2018)
33	LRP1	In vivo- human	ELISA	The level reduced in AD	CSF/ plasma	(Shinohara et al., 2014)
34	sRAGE	In vivo-human	Quantikine sandwich ELISA kits (R&D Systems, Minneapolis, MN, USA)	Level dwindled in AD compared with the normal group	plasma	(K. Li et al., 2010)
35	visinin-like protein-1 (VILIP-1)	In vivo-human	microparticle-based immunoassay (Erenna, Singulex, USA) analyzed in CSF and blood	The level increased	CSF	(R. Tarawneh et al., 2011)
36	neurogranin (Ng)	In vivo-human	in-house immunoassay for Ng	Increased Associate with Aβ	CSF	(N. Mattsson, Insel, Palmqvist, Stomrud, et al., 2016)
37	synaptosome–associated protein-25 (SNAP-25)	In vivo- human	Erenna® immunoassay assay	The level increased in AD patients in the early stage	CSF	(H. Zhang et al., 2018)
38	synaptotagmin-1 (Syt-1)	In vivo- human	mass spectrometry-based assay	The level was significantly elevated in the MCI group in comparison to AD and normal	CSF	(Öhrfelt et al., 2016)
39	growth-associated protein 43 (GAP-43)	In vivo- human	ELISA	The level increased and was associated with synaptic degeneration	CSF	(H. Zhang et al., 2022)
40	Galectin-3 (Gal-3)	In vivo- human	Immunohistochemistry	Increased in the CSF and associated with Aβ level	CSF	(Boza-Serrano et al., 2022)
41	kallikrein 6	In vivo-human	mass spectrometry	Increased in the CSF of AD	CSF	(Giannisis et al., 2022)
42	glial fibrillary acidic protein (GFAP)	In vivo-human	Neurology 2-Plex B kit from Quanterix	Increased in plasma and CSF in MCI and with higher intensity in AD	CSF/ plasma	(Parvizi et al., 2022)
43	Ig mμ chain C region (IGHM)	In vivo- human	Western blot	Increased in AD	plasma	(Song et al., 2014)
44	Hexacosanoic Acid (C26:0)	In vivo- human	gas chromatography	Increased in AD	plasma	(Zarrouk et al., 2015)
45	24S-Hydroxycholesterol	review	3 studies from 2000 to 2009	Negatively correlated with AD severity	plasma	(Zarrouk et al., 2014)
46	7-Ketocholesterol	review	5 studies from 2014 to 2018	Increased in the plasma of neurodegenerative diseases	plasma	(Mahalakshmi et al., 2021)
47	7β-hydroxycholesterol, 25-hyroxycholesterol, and 27-hydroxycholesterol	In vivo- human	gas chromatography and mass spectrometry	Increased in the plasma of neurodegenerative diseases	plasma	(Zarrouk et al., 2020)

### 3.2 Signaling pathway of biomarkers

Biomarkers' role can be classified into multiple subgroups. However, in this study, there was an attempt to minimize the number of subgroups. Hence, the number of groups decreases to 4 major domains of inflammation, synaptic and axonal dysfunction, amyloidogenic, and mitochondrial dysfunction.

#### 3.2.1 Inflammation

From the inflammation aspect, the role of biomarkers and their signaling pathway is briefly mentioned. Macrophage inflammatory protein 1-alpha (MIP-1α) is expressed in the endothelial cells of the Blood-brain barrier (BBB) and influenced by oxidative stress. Elevation of oxidative stress mediators like hydrogen peroxide, or enhancement in the concentration of the lipopolysaccharide, had a dose-dependent effect on in vitro MIP-1α upregulation (Tripathy et al., 2007). Similar to MIP-1α, Angiopoietin-2 (ANG-2) is mainly expressed in pericytes (Reiss et al., 2015), and regulates the permeability of the BBB (Gurnik et al., 2016). Moreover, this biomarker is reported to increase in inflammatory status of the brain (Scholz et al., 2011). In contrast, the high level of brain natriuretic peptide (BNP) has a negative correlation with endothelial function, resulting in the appearance of cerebrovascular disease (Gurnik et al., 2016). In addition, recombinant human brain natriuretic peptide (rhBNP) has an inhibitory role toward nuclear factor kappa B (NF-κB) and mitogen-activated protein kinase (MAPK) pathways, and in the inflammatory condition, it regulates Interleukin-10 (IL-10), IL-6, and Tumor necrosis factor alpha (TNF-α) (X. Li et al., 2018). Besides inflammatory trigger biomarkers, Complement factors have a protective role in inflammation and infection along with maintaining homeostasis across the brain (Hammad et al., 2018). Moreover, The utmost important biomarker for astrocytes, named Glial fibrillary acidic protein (GFAP), upregulates AD with an inhibitory effect on inflammation, after nerve injury (S. Zhang et al., 2017). While Galectin-3 (GAL-3) is expressed in the immune and inflammatory mediated cells (F.T. Liu & Hsu, 2007), PGRN has a remarkable expression in inflammatory conditions (C. Liu et al., 2020). Unlike the mentioned biomarkers, HFABP is primarily a biomarker of cardiovascular disease. Nevertheless, its level alternation indicates fibrosis, chronic inflammation, ischemia, and angiogenesis (Rezar et al., 2020).

#### 3.2.2 Synaptic and axonal dysfunction

Epidermal Growth Factor Receptor (EGFR) is mainly involved in kidney chronic inflammation as a tyrosine kinase receptor (Rayego-Mateos et al., 2018). However, it has been conceptualized to participate in calcium influx. Therefore, synaptic dysfunction and disability of neurotransmitter release can be reflected through EGFR (Delos Santos et al., 2017). β-2-glycoprotein is involved in the coagulation pathway (Miyakis et al., 2004), vascular inflammation, and neural clearance, mediated by phagocytosis (Grossi et al., 2021). Hence, alternation in its level can reflect the status of neural damage in the brain. Insulin-like growth factor binding protein-2 (IGFBP-2) is involved in cellular signaling as a CNS growth factor, visualizing synaptic status and cognitive state (Khan, 2019). Neuron-specific enolase (NSE) is expressed specifically in neuroendocrine cells and neurons, as an acidic protease. The level of this biomarker is the most potential illustrator of nerve injury (Miao et al., 2020). Ng is highly expressed in the hippocampus, cortex, amygdala, and striatum neurons, as a postsynaptic protein (Szabo & Nemeroff, 2015). The visinin-like proteins VILIP-1 and VILIP-3, are two compounds that mediated neuronal calcium channel proteins as well as dendritic growth and cyclic nucleotide signaling. These compounds have downregulation compared to cognitively normal subjects and their level is correlated with AD stages and symptoms (Braunewell, 2012). Among post-synaptic proteins, there are two potential biomarkers named α-Synuclein and GAP-43. The first one has inhibitory properties in transferring signals, when neurotransmitters are released in abundance, on the synaptic cleft (Bendor et al., 2013), while the second one participates in neural growth and neural plasticity as well as axonal regeneration triggered by nerve injury (Rosskothen-Kuhl & Illing, 2014). Kallikrein 6, which is classified as a serine protease, mediates neurodegeneration and induces glutamate neurotoxicity (Yoon et al., 2013). Virus-like particles-1 (VLP-1) is recognized as a structure that consists of particles similar to viruses. Up to this date, there is inadequate data about the role of these particles in the brain, and researchers just focus on drug delivery using these particles (Nooraei et al., 2021).

#### 3.2.3 Amyloidogenic

Amyloidogenic biomarkers are discussed in the Aβ section. Here are supplementary biomarkers related to the Aβ signaling pathway: LDL receptor-related protein 1 (LRP1) is another CSF and blood biomarker that has the potential to differentiate two stages of AD classified as MCI and late-stage-AD. Sultana et al. and Shinohara et al. reached the same result in which a low level of LRP1 was associated with AD (Shinohara et al., 2014, Sultana et al., 2010). Moreover, the level of Cortical LRP1 also depicts the same result of the CSF/ blood concentration, and the pattern of dwindled LRP1 is equivalent to Aβ accumulation (Shinohara et al., 2014). Similar to Aβ, LRP1 has several forms; one of them is known as Oxidized sLRP1, and its level is thought to be increased in MCI and AD patients (Sagare et al., 2011). Another biomarker in AD that can be detected in the CSF and blood is named Soluble Receptor for Advanced Glycation End Products (sRAGE). Similar to the LRP1, the amount of the sRAGE negatively correlates with AD stages in a way that the normal group demonstrated the highest concentration of this biomarker. This fact has been justified in three different original articles (Emanuele et al., 2005, Ghidoni et al., 2008; K. Li et al., 2010). Fibrinogen γ chain is reported to mediate factor XIII and fibrin polymerization and is hypothesized to be involved in vascular dementia and interaction with Aβ accumulation (Bian et al., 2021, Lee et al., 2007). Pancreatic Polypeptide (PP) is conceptualized to be comorbid with AD appearance, either in association with type 2 diabetes or without it (Roberts et al., 2015). Its role is pronounced in the obese population since it takes part in appetite suppression and energy intake (Holzer et al., 2012). Eventually, α2 M binds to proteins and participates in the elimination of pathogenic particles as a humeral immune system (Vandooren & Itoh, 2021). It is claimed that α2 M interacts with LRP1 in the elimination and transportation of Aβ (Song et al., 2014).

#### 3.2.4 Oxidative stress

One of the pathophysiology pathways of AD was mentioned as oxidative stress. Oxidative stress was suggested to be initiated by aggregation of the Aβ, inflammatory molecules, and lipid peroxidation products (Gamba et al., 2011), in which lipid peroxidation compounds had a sensitivity of 81.3 %, specificity of 64 %, and accuracy of 75.3 % in the differential diagnosis of AD (Ferré-González et al., 2022). Besides, neuroinflammation-induced molecules were a candidate for involvement in or deteriorating oxidative stress, among which oxysterol was proposed to be involved as a member of cholesterol metabolism in the brain. Two cholesterol derivations named 27-hydroxycholesterol (27-OHC) and 24S-hydroxycholesterol (24S-OHC) were involved in the pathology of AD by regulating immune responses and inflammatory factors (Testa et al., 2016). In addition, 7-ketocholesterol was stated to be involved in membrane permeability through interacting with lipid rafts and participated in oxidative stress in AD (Mahalakshmi et al., 2021).

#### 3.2.5 Mitochondrial dysfunction

Afamin as a protein involved in vitamin E transport is downregulated in AD. It is expressed in BBB endothelial cells and participates in mediating oxidative stress (Kratzer et al., 2009). Moreover, afamin and Ig mμ chain C region (IGHM) are suggested to participate in acute inflammation (Song et al., 2014). Hemopexin as an antioxidant interacts with extracellular heme and prevents neurotoxicity (Latunde-Dada, 2016). It also regulates reactive oxygen species (ROS) and protects neurons against oxidative stress (Hahl et al., 2013) (Fig. 2).

### 3.3 Neuroimaging biomarkers

#### 3.3.1 Conventional MRI

Neuroimaging biomarkers are primarily defined as utilizing neuroimaging techniques to evaluate the specific hallmark of the disease. For instance, by using conventional MRI, atrophy in different regions of the brain could be detected (Heidari et al., 2020, Heidari et al., 2017a, Heidari et al., 2017b, Heidari et al., 2023), especially in the medial temporal lobes, to distinguish AD (H. Wang et al., 2011). This method is widely implemented in clinical diagnosis, in spite of less accuracy in early detection of AD (Murray et al., 2014). Besides conventional methods of MRI, including T1 and T2 weighted images, there are more sequences that could provide detailed information about the disease stage. One of these methods is MRS, which is mostly utilized in research approaches.

#### 3.3.2 Magnetic resonance spectroscopy (MRS)

MRI acquisition provides information on neural shrinkage, structural integrity, and volume of white matter, while MRS makes it possible to be aware of metabolite changes, similar to data provided in PET. One of the hallmarks of AD is the level of N-acetyl aspartate (NAA), which is negatively correlated with neuritic plaques (Modrego & Fayed, 2011). The use of MRS in the detection of the early stages of AD is still under consideration. In Kantarci et al. research, the ratio of NAA to creatine (Cr) and NAA to mI (myoinositol) was applied to diagnose the early stage of AD, with the aid of ^1^H MRS. These ratios are reported to be associated with Aβ accumulation and synaptic loss (Kantarci & Jicha, 2019). The regional changes of metabolites in the brain, especially in the temporal area, were asserted to be helpful in distinguishing AD from normal participants (Sheikh-Bahaei, 2020). Nevertheless, to this date, the role of MRS in clinical diagnosis is nebulous due to inadequate data on the pathological pathways of AD. Since neuronal integrity and membrane composition are disrupted in AD, the level of NAA and choline (Cho) can be monitored as a biomarker in AD. Other compounds suggested to be changed during AD, are Cr, phosphocreatine (PCr), mI, glutamate, and γ-aminobutyric acid. The last two are assessed to track synaptic dysfunction mediated by neurotransmitters (Kehoe et al., 2014). To provide a more complex conclusion, instead of considering metabolites solely, it can be possible to measure the NAA/Cr and NAA/mI ratio in which low values are correlated with neurofibrillary tangle, while the increased rate of mI/Cr and decreased rate of NAA/mI is correlated with Aβ accumulation. Moreover, in the early stages of AD, it is possible to predict synaptic loss by considering a reduction in NAA/Cr (Murray et al., 2014). However, because the application of MRS needs further research to be accepted in clinical diagnosis, researchers implement a combination of methods along with MRS to evaluate AD patients. In this case, a complex series of data in correlation with the patient's condition is provided (Mohtasib et al., 2022).

#### 3.3.3 Positron emission tomography (PET) scan

As mentioned before, one of the techniques to evaluate compound concentration in the brain is PET scan. This method has a wider range of targets in comparison with MRS since radioligands can be developed to assess either static membrane receptors or dynamic enzymes and proteins (Chandra et al., 2019). The form of radioisotopes in the detection of tau protein, are listed as [^18^F]PI2620, [^11^C]PBB3, [^18^F]GTP1, [^18^F]PM-PBB3, [^18^F]MK6240, [^18^F]RO69558948, [^18^F]THK5117(5317), [^18^F]T808, and [^18^F]THK5351. In contrast to tau, Aβ has a limited number of radioisotopes to be detected through PET scan, named [^18^F]NAV4694, [^11^C]PiB, [^18^F]Flutemetamol, [^18^F]Florbetaben, and [^18^F]Florbetapir (Andersen et al., 2021). One of the well-known limitations of PET scans is the lack of distinction in diagnosing one disease from the other. From the results of Shimada et al., it could be perceived that PET scan is inadequate to discriminate Lewy Body Disease from AD since the Aβ deposition pattern is similar in both of them (Shimada et al., 2013). However, developing more specific radioactive compounds may facilitate the diagnosis procedure. In this regard, Fleisher et al. and Andersen et al. highlighted [^18^F]-Flortaucipir (AV1451) in the highest sensitivity, but a low specificity in recognition of AD, based on tau protein (Andersen et al., 2021, Fleisher et al., 2020).

#### 3.3.4 Diffusion tensor imaging (DTI)

Although DTI techniques have less application in AD, their parameters could anticipate the disease as well as the stage of it. Traditional techniques were based on the estimated condition of atrophy in different segments of the brain while utilizing the DTI method provides quantitative information on the disease status. In this case, the disease progression becomes more pronounced, since the numeric values could be compared with managerial competence versus descriptive information. In this regard, analyzing the data from axial diffusivity of the gray matter (GMAxD) could compensate for MRI data acquisition of hippocampus atrophy (Mohtasib et al., 2022). Although GM has long been evaluated to determine AD, nowadays white matter has been brought into focus, which also depicts a downward trend in volume over time. A specific part of white matter named superficial white matter (SWM), has a high vulnerability to AD progression. With the aid of this parameter, three groups of AD, MCI, and normal individuals, are differentiated from each other (Bigham, Zamanpour, & Zare, 2022). It can be also possible to limit our data to specific sections of the brain, by modifying the region of interest (ROI). The areas in which researchers found differences between normal, MCI, and AD groups, are listed as the left tapetum, splenium of the corpus callosum (CC), left fornix (crus)/stria terminalis and left hippocampal section of the cingulum. These areas all have significant alternation in the diffusivity parameters, except for fractional anisotropy (FA). Moreover, the last area mentioned in the list has the potential to distinguish the early and late stages of the MCI, since based on voxel-based analyses (VBA), this area has a remarkable elevation in diffusivity parameters of the DTI (Nir et al., 2013). Eventually, several studies reported limitations in the detection of AD through DTI and recommended combining MRI techniques to provide a more accurate picture of what exactly happens in AD patients. Accordingly, a combination of three methods of structural MRI (sMRI), [11 C]-Pittsburgh Compound B (PiB) PET scan, and DTI, provides enormous data across the AD stage (Agostinho et al., 2021). Moreover, the combination of resting state functional MRI (rsfMRI) and DTI to measure functional connectivity (rsfMRI-FC) and structural connectivity (DTI-SC) respectively, depicts a high correlation with clinical symptoms along with cognitive assessments scores, including Mini-Mental State Examination (MMSE) with negative correlation, Clinical Dementia Rating scale Sum of Boxes (CDR-SB) and Functional Activities Questionnaire (FAQ), that both have a positive correlation. Nonetheless, there is no detectable correlation with the Montreal Cognitive Assessment (MoCA) (Xu, Xu, Han, & Yao, 2022).

#### 3.3.5 Diffusion-weighted imaging (DWI)

Another method in AD detection and determination of its stage is DWI. There are inadequate studies on DWI solely in AD patients, due to researchers’ tendency to implement their main results based on other MRI sequences and the use of DWI as a supplementary technique. In Mak et al., a combination of neuroimaging techniques was executed to predict AD progression. In this research, 3D T_1_-weighted images, DTI, and DWI were utilized. The results revealed a remarkable WM destruction justified by diffusion parameters including FA, mean diffusivity (MD), and radial diffusivity (RD), while there was not any detectable gray matter atrophy. The MD and RD have elevated values in contrast to FA. Moreover, it is possible to debate whether AD patients carry an APOE-ε4 allele, without performing the genetic test for patients with APOE-ε4 status, which depicted hyperperfusion condition along with early dementia onset (Mak et al., 2021).

#### 3.3.6 Diffusion tensor tractography (DTT)

**Fig. 3:**
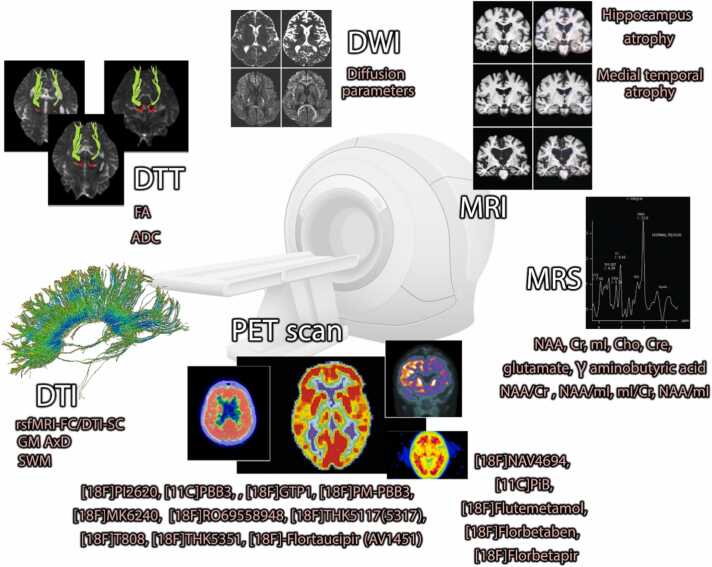
Neuroimaging biomarkers of conventional and advanced MRI techniques, as well as PET scan in AD. MRS and PET scans had the highest number of markers dedicated to AD, while there were few data suggesting the usefulness of the fMRI technique in AD detection or differentiating different stages of it.

One of the challenges in the acquisition of DTI data is the ROI issue which depicts instability in placement and the other may be subjected to fiber contamination. Moreover, isolating WM tracks decreases artificial disruption caused by large data processing (McCreary et al., 2020, Raposo et al., 2021). In this regard, DTT was applied to the visualized complex network connections of WM tracks. In Morikawa et al. research, dTV II software was employed to determine the WM tracts from DTI data. The results of the study illustrated an association between FA values and the score of the cognitive assessment, including MMSE with a positive correlation and the Alzheimer's Disease Assessment Scale-cognitive component-Japanese version (ADAS-Jcog), with a negative correlation. Furthermore, the apparent diffusion coefficient (ADC) has an inverse correlation with the mentioned assessment, compared to FA (Morikawa et al., 2010) (Table 2) (Fig. 3).

**Table 2:** Neuroimaging biomarkers in AD.

Method	Hallmark	Procedure	Defined	Study
MRI	Medial temporal atrophy	Multimodal data	Neuron loss	(H. Wang et al., 2011)
	Hippocampus atrophy	National Institute on Aging and the Alzheimer's Association	Neuron loss	(Albert et al., 2011)
MRS	N-acetyl aspartate (NAA)	1.5 T	negatively correlated with neuritic plaques	(Modrego & Fayed, 2011)
		NL:16,		
		MCI:32,		
		AD:16,		
		TR: 560 ms, TE: 12 ms, 90 flip		
		angle, number of excitations: 1, matrix size 256 ×160, FOV: 24× 24 cm, slice thickness/gap		
		5/0 mm) was obtained to locate a 2 ×2×2 cm voxel		
	creatine (Cr)	A narrative review of 6 study	Cell energy consumption	(Kehoe et al., 2014)
	Myoinositol (mI)	A narrative review of 6 study	Glia growth	(Kehoe et al., 2014)
	choline (Cho)	A narrative review of 6 study	Membrane composition	(Kehoe et al., 2014)
	phosphocreatine (Cre)	A narrative review of 6 study	Cell energy consumption	(Kehoe et al., 2014)
	glutamate	A narrative review of 6 study	Excitatory neurotransmitter	(Kehoe et al., 2014)
	γ aminobutyric acid	A narrative review of 6 study	Inhibitory neurotransmitter	(Kehoe et al., 2014)
^1^H-MRS	NAA/Cr	3 T	Associated with AD symptoms and dementia	(Murray et al., 2014)
		41 autopsied individuals		
		TR = 2000 ms, TE = 30 ms, 2048 data points, and 128 excitations		
	NAA/mI	3 T	Associated with AD symptoms and dementia	(Murray et al., 2014)
		41 autopsied individuals		
		TR = 2000 ms, TE = 30 ms, 2048 data points, and 128 excitations		
	mI/Cr	3 T	Its increased value correlated with Aβ accumulation	(Murray et al., 2014)
		41 autopsied individuals		
		TR = 2000 ms, TE = 30 ms, 2048 data points, and 128 excitations		
	NAA/mI	3 T	Its decreased value correlated with Aβ accumulation	(Murray et al., 2014)
		41 autopsied individuals		
		TR = 2000 ms, TE = 30 ms, 2048 data points, and 128 excitations		
PET	[18 F]PI2620, [11 C]PBB3, [18 F]GTP1, [18 F]PM-PBB3, [18 F]MK6240, [18 F]RO69558948, [18 F]THK5117(5317), [18 F]T808, [18 F]THK5351, and [18 F]-Flortaucipir (AV1451)	Meta-analysis	Determine tau level and accumulation pattern	(Andersen et al., 2021)
		27 randomized clinical trial		
	[18 F]NAV4694, [11 C]PiB, [18 F]Flutemetamol, [18 F]Florbetaben, and [18 F]Florbetapir	Meta-analysis	Determine Aβ level and accumulation pattern	(Andersen et al., 2021)
		27 randomized clinical trial		
DTI & fMRI	rsfMRI-FC/DTI-SC	3 T,	Correlates with multiple cognitive assessments and reflects symptom status	(Xu et al., 2022)
		NL: 62,		
		MCI: 31,		
		AD: 19		
		rsfMRI: EPIS sequence		
		FA: 90°; TR: 3000 ms; TE: 30 ms; ST: 3.4 mm;PS: 3.4 × 3.4 mm^2^; MS: 448 × 448; TP:197		
		DTI: GD: 54 (94 cases), 30 (18 cases); FA: 90°; TR: 7200/12, 400 ms; TE: 56/95 ms; ST: 2.0 mm; pixel size: 2.0 × 2.0 mm^2^; matrix size: 1044 × 1044		
DTI	GM AxD	3 T,	Reflect brain atrophy	(Mohtasib et al., 2022)
		NL:20,		
		AD,20		
		multi-shell HARDI,		
		TR: 7800 ms, TE: 100 ms, FA: 90°, voxel size: 2.4 × 2.4 × 2.4 mm^3^		
	SWM	3 T	Differentiate AD and MCI patients from the normal group	(Bigham et al., 2022)
		NL: 24,		
		MCI: 24,		
		AD: 24,		
		TR: 13000 ms, TE: 68.3 ms, FA: 90°, field strength: 3.0, ST: 2.7 mm		
DWI	Diffusion parameters	3 T	Correlate with cognitive tests	(Mak et al., 2021)
		Without ε4 gene: 42,		
		With ε4 gene: 37,		
		b = 1000 s/mm^2^		
		TR = 11.7 s, TE = 90 ms, 63 slices, voxel size = 2 × 2 × 2 mm, flip angle = 90°)		
DTI & DTT	Fractional Anisotropy & ADC	1.5 T	Correlate with cognitive tests	(Morikawa et al., 2010)
		AD: 30,		
		TR: 2300 ms,		
		TE: 122 ms,		
		B: 1000 s/mm^2^,		
		6-axis encoding,		
		FOV: 230 mm,		
		Matrix: 128 × 128,		
		slice spacing: 3 mm,		
		slice thickness: 3 mm, averaging: 6		
		dTV II and Volume-One 1.72 software		

NL: normal, MCI: mild cognitive impairment, AD: Alzheimer’s disease, FA: flip angle, TR: time of repetition, TE: time of echo, ST: slice thickness, FOV: field of view, PS: pixel spacing, MS: matrix size, TP: time points, EPIS: echo planar imaging, GD: gradient directions, ST: slice thickness, GM: gray matter, AxD: axial diffusivity, T: tesla, SWM: Superficial white matter, ADC: apparent diffusion coefficient, DTI: diffusion tensor imaging, DTT: diffusion tensor tractography, DWI: diffusion weighted imaging

### 3.4 Interactions between biomarkers in AD

AD is a complex disorder in which pathophysiological abnormalities, detectable in vivo by biomarkers, precede overt clinical symptoms by many years to decades. Biomarkers are measurable indicators of what’s happening in the body, and they can be found in blood, other body fluids, organs, and tissues. The current AD diagnostic methods are typically invasive and expensive, limiting their potential for widespread use. However, the development of biomarkers in available biofluids, in combination with other risk factors, will provide a novel solution that may revolutionize the early diagnosis of AD (Gunes et al., 2022). Current AD biomarkers fall into two categories: biomarkers of amyloid-β plaques and tau-related neurodegeneration (Jack & Holtzman, 2013). The most widely used CSF biomarkers for AD measure beta-amyloid 42, tau, and phosphorylated tau. The updated research framework for AD, proposed by the National Institute of Aging-Alzheimer’s Association (NIA-AA) Work Group, emphasizes the importance of incorporating biomarkers in AD research and defines AD as a biological tau, amyloid, and neurodegeneration (ATN) entity (Rawan Tarawneh, 2020).

Several studies have investigated the potential interactions between different CSF and plasma biomarkers and neuroimaging biomarkers in the context of AD. These interactions are important for understanding the complex nature of AD pathophysiology.

One study compared plasma and CSF markers in predicting cognitive decline and found that for studies of moderate length, either plasma or CSF markers predict changes of similar magnitude. The study demonstrated that both plasma and CSF measures of Aβ42/Aβ40 and NfL independently predicted a decline in a global cognitive composite (Aschenbrenner et al., 2022). Another study emphasized the potential utility of composite biomarkers as suitable outcome measures for AD and the need for biomarkers that allow for early diagnosis and intervention (Márquez & Yassa, 2019). Furthermore, a study analyzed the evolution of 13 key CSF and plasma biomarkers about increasing Aβ accumulation during AD. The study found that plasma and CSF biomarkers changed approximately at the same point, with plasma biomarkers having smaller dynamic ranges (Palmqvist et al., 2019). Neuroimaging biomarkers have also been the focus of research, with numerous advances made in developing biomarkers for AD using neuroimaging approaches. These approaches offer versatility in understanding and targeting various aspects of AD-related brain changes (Márquez & Yassa, 2019). In summary, the research indicates that both CSF and plasma biomarkers, as well as neuroimaging biomarkers, play a crucial role in understanding the pathophysiology of AD. The studies highlight the potential interactions between these biomarkers and their implications for the development of accurate, comprehensive models of AD biomarker evolution.

### 3.5 Therapeutic prospects

#### 3.5.1 Determination of the AD stage

Traditional clinical methods of detecting AD are based on the higher levels of Aβ and tau protein, as well as atrophy in the brain. Nevertheless, it has been reported that AD may be concomitant, without Aβ elevation in CSF and blood. In this case, plasma glial fibrillary acidic protein (GFAP) and NFL, discriminate between AD patients with or without Aβ enhancement. Moreover, the result of cross-sectional research reveals the superiority of GFAP versus NFL in differentiating AD from MCI and normal group (Parvizi et al., 2022). However, the NFL level demonstrates neurodegenerative diseases, independently from Aβ accumulation. Similar to NFL, NG increase has the same consequence (N. Mattsson et al., 2016). In addition, the growth-associated protein 43 (GAP-43) CSF level, provides a wider concept of the state of AD due to its correlation with Aβ, p-tau, t-tau, and even atrophy occurring in the hippocampus. Moreover, hypometabolism, which is monitored by PET scan, is reported to be associated with the GAP-43. Hence, this biomarker is suggested to be implemented in clinical diagnosis and determination of the intensity of the disease (H. Zhang et al., 2022). One of the main challenges in AD detection is its uncertainty in the early stage of the disease, in which neuroimaging methods do not reflect any significant changes in comparison to the normal group. In this situation, sTREM2 and PGRN which are also correlated with the level of p-tau and t-tau, could be useful in AD diagnosis (Morenas-Rodríguez et al., 2019). This fact reveals the neuroinflammatory role in the onset of AD symptoms (Boza-Serrano et al., 2022). Among neuroinflammatory biomarkers, the YKL-40 level is not a potential biomarker in the specific detection of AD. While the level increases significantly, it has been claimed as the same status in Lewy bodies dementia (DLB), frontotemporal dementia, and vascular dementia (VaD) (Hao et al., 2022). Moreover, the sTREM2 has no remarkable increase in the AD group, compared to normal cases (Morenas-Rodríguez et al., 2019). Finally, the most complicated part of AD stage determination is differentiating AD from MCI, in which most of the clinical symptoms overlap with each other. One of the biomarkers that could perfectly discriminate these stages is synaptotagmin-1 (Syt-1) (Öhrfelt et al., 2016).

#### 3.5.2 Evaluation of the effectiveness of the novel drugs

Since Ng level changes in accordance with the progression of the disease, it can be implemented in following up on drug efficiency (Hao et al., 2022). One major point in the disease progression is neuroglia function. Due to changes in the number of neuroglia markers in comparison to the baseline in the normal participants, the mechanism of the novel drugs in influencing AD is suggested to be evaluated through Galectin-3 (Gal-3), which is a beta-galactosidase binding protein used as a biomarker of microglial function (Boza-Serrano et al., 2022). Furthermore, the inflammation procedure in AD could be better monitored with YKL-40 (chitinase-3 like-1, human cartilage glycoprotein-39, and chondrex), as a biomarker of astrocytic inflammation (Giannisis et al., 2022). Its concentration in CSF is higher than other neuroglia biomarkers, which facilitates the evaluation of the CSF through the ELISA kit. In AD patients who carry mutant genes, one of the important factors to be evaluated is the level of Apolipoprotein E (apoE). This factor is responsible for the majority of symptoms onset, without reflecting a significant association with neuroglia biomarkers like YKL40 (Giannisis et al., 2022, Wu et al., 2022). Two ways are available for measuring this biomarker. Firstly, the amount can be determined directly in the plasma of the patients, through ELISA kits. Secondly, the level can be predicted indirectly, through other biomarkers including α-synuclein and kallikrein 6, which are negatively correlated with apoE. While the first method seems more convenient to implement, the plasma level of apoE depicts a fluctuation pattern without exactly reflecting the amount in the CNS. Hence, measuring the amount indirectly could compensate for uncertainty caused during the direct assessment. Nevertheless, there are still several challenges in the determination of the level of these two associated biomarkers, like unavailable commercial kits (Giannisis et al., 2022).

Inflammation is one of the major factors in the onset of AD symptoms. Hence, measuring neuroinflammatory markers secreted from microglial cells reflects the efficiency of suggested drugs in AD. Neuroinflammatory factors including GFAP, sTREM-2, YKL-40, and Galectin-3 (Gal-3), can be measured in the CSF. According to the results of the studies, all of them are upregulated in the brain. However, Gal-3 is suggested to be evaluated in the drug efficiency assessment, since its expression is remarkable in microglia, and its association with Aβ accumulation, has been mentioned in both human and animal research (Boza-Serrano et al., 2022). Besides the inflammation aspect, neuroimaging methods are recommended to evaluate the rate and development of atrophy. Nevertheless, long-term image acquisition for drug follow-up accompanies a higher research budget. Therefore, the SNAP-25/Aβ42 ratio is utilized to track atrophy in the early stage of MCI. The elevated ratio demonstrates the progression of the disease, along with the development of cognitive impairment. Moreover, measuring the CSF level of SNAP-25 or SNAP-25/Aβ42 has a high specificity in detecting MCI and AD progression (H. Zhang et al., 2018). In a clinical trial to assess the efficiency of a new medication named CT1812 for AD, fluorodeoxyglucose (FDG) PET, synaptic vesicle glycoprotein 2 A (SV2A), and volumetric MRI were utilized to measure the changes over 24 weeks (van Dyck et al., 2024). In another clinical trial, the efficacy of the KHK6640 was explored by measuring the level of PET glucose metabolism and CSF tau level (Cantillon et al., 2024).

#### 3.5.3 Role of artificial intelligence (AI)

Since analyzing vast amounts of data produced by MRI systems by current signal and image processing, associated with covert bias and concluded based on human results, consists of inaccuracy decisions, artificial intelligence can be used to manage of mentioned big data in clinical research. One of its applications is disease diagnosis in which the unknown data can be evaluated by providing verified data of disease condition versus normal condition, leading to providing a proper prognosis. In recent research conducted by Ushizima et al., by using IHCNet, MRI data could be analyzed to provide a better picture of disease status, with an algorithm based on molecular and imaging data (Ushizima et al., 2022). In another research, an application named BAAD was developed to distinguish the diseases by utilizing neuroimaging data. The result of the study indicated a significant rate of accuracy and specificity, ranging from 88 % to 100 %, compared to radiology experts with a range of 57.5–70 %. In the mentioned research, two subunits were developed in two domains of “brain structure” and “cognitive state”, listed as support vector machine structure (SVMst), and support vector machine cognitive (SVMcog). The latter is based on the Mini-Mental State Examination score (MMSE). With the aid of this software, the MCI progression can be tracked. Hence, it is useful to assess the drug effect over time, with the lowest bias (Syaifullah et al., 2020). In a cohort study, a random forest algorithm was applied using serum proteomic data of the MCI and normal participants resulting in an AUC of 0.881 in distinguishing patients and normal cases. Accordingly, the top key serum biomarkers dedicated to high accuracy were nominated as P14780-MMP9, P0C0L5-C4B, Q13201-MMRN1, and Q9NZP8-C1RL (Tao et al., 2024). In addition, the exact level of Aβ40, Aβ42, P-tau217, P-tau181, and NFL in femtomolar level was determined to be used as an input of the machine learning algorithm to differentiate different stages of AD, resulting in an AUC of 0.94 (S. Wang et al., 2024). Besides, using plasma and CSF biomarkers in combination boosts the accuracy of the machine-learning algorithms. Accordingly, it was reported that the AUC of 0.94 using the SVM algorithm and plasma biomarkers as data enhanced to 0.98 by adding CSF biomarkers data (Luz et al., 2023).

Among the limitations of the included studies, bias in selecting patients' disease manifestation could be mentioned, in which due to applying multiple exclusion criteria, patients with poor prognosis or in the late stage were not involved in the study (Tijms et al., 2018). In addition, the limited sample size was reported as the main limitation of some of the studies (Boza-Serrano et al., 2022, Giannisis et al., 2022, Hampel et al., 2018). Another limitation in the meta-analysis of the novel biomarkers in AD was heterogeneity among studies due to the low number of studies and controversies in the results (Olsson et al., 2016). Moreover, the lack of study on the pathophysiological signaling pathway of some biomarkers including PP, despite the high correlation between the biomarker and disease, the conclusion on the link of the biomarker to disease is uncertain (Rehiman et al., 2020). In addition, the lack of longitudinal analysis for APP-α and APP-β levels was suggested as one of the factors in which these biomarkers did not reveal significant differences in normal and AD groups in comparison to other biomarkers (Kettunen et al., 2022). Eventually, one of the limitations of the imaging techniques was low specificity for AD disease (Andersen et al., 2021).

## 4 Conclusion

Among various fluid biomarkers, it can be concluded that CSF levels of biomarkers have a higher sensitivity in comparison to blood ones. For diagnostic purposes, it is better to consider multiple biomarkers rather than focusing on a specific one. In this regard, a decrease in the CSF levels of Aβ42, coinciding with the increased levels of T-tau and P-tau, predicts AD (Tan et al., 2014). Nevertheless, the plasma and serum levels of the well-known biomarker of AD named as Aβ, primarily reflect the existence of senile plaque rather than AD-related Aβ accumulation (Olsson et al., 2016). Moreover, the application of Syt-1 among other CSF/blood biomarkers, has the highest sensitivity to the specified MCI group. Besides, imaging techniques have the highest sensitivity in detecting and discriminating AD, especially when they are used with more than one MRI sequence; for instance, the combination of DTI and DTT or fMRI and DTI. Finally, to minimize the procedure bias caused by either humans or equipment, the AI method is widely developed to compensate for imaging errors and facilitate the conclusion, based on neuroimaging results. Moreover, from the cost efficacy aspect, selecting plasma biomarkers to detect or monitor AD is a better option than CSF or neuroimaging biomarkers.

As you know, AD is one of the most common neurodegenerative disorders with cognitive impairment, which threatens the lives of millions of people, especially the elderly, worldwide. AD is currently incurable due to the absence of a timely diagnosis and effective intervention strategy. Thus, there is a strong desire for innovative diagnostic and therapeutic methods. In recent years, there has been increasing focus on the role of biomarkers in understanding the development, early detection, and treatment of diseases. However, the specific contribution of biomarkers to the development and treatment of AD has not been fully elucidated (Mumtaz et al., 2022; Hui Wang et al., 2023). Biomarkers for AD are intended to aid in early prognosis and tracking disease progression, potentially enabling the design of disease-modifying therapies (Mumtaz et al., 2022). It would create opportunities to broaden the use of fluid/neuroimaging biomarkers in both fundamental investigation and theranostics of AD in clinical settings. In the future, the careful utilization of biomarker testing will be crucial for the precise identification of patients eligible for disease-modifying treatments. In addition, it will be vital to develop new biomarkers that minimize the need for invasive procedures and enhance the more accurate detection of specific pathological processes.

## Ethics Approval

This study was conducted under the supervision of the Qazvin University of Medical Sciences (QUMS) with the ethical code IR.QUMS.REC.1400.494.

## Funding

This research did not receive any specific grant from funding agencies in the public, commercial, or not-for-profit sectors.

## CRediT authorship contribution statement

A. Rustamzadeh and Z. Ghobadi contribute to the study's conception, design, and data interpretation. A. Ariaei and H. Mohammadi and E.A Charkhat Gorgich contributed to data collection, writing, and editing of the manuscript. All authors commented on previous versions of the manuscript. All authors read and approved the final manuscript.
